# ‘Herbals she peruseth’: reading medicine in early modern England

**DOI:** 10.1111/rest.12079

**Published:** 2014-08-05

**Authors:** Elaine Leong

**Affiliations:** Max Planck Institute for the History of Science

**Keywords:** gender, history of reading, households, manuscript culture, medicine and health

## Abstract

In 1631, Richard Brathwaite penned a conduct manual for ‘English Gentlewomen’. In Brathwaite's mind, the ideal English gentlewoman was not only chaste, modest and honourable but also an avid reader. In fact, Brathwaite specifically recommends English gentlewomen to first peruse herbals and then to deepen their medical knowledge via conference. Centred on the manuscript notebooks of two late seventeenth-century women, Margaret Boscawen (d. 1688) and Elizabeth Freke (1642–1714), this article explores women and ‘medical reading’ in early modern England. It first demonstrates that whilst both women consulted herbals by contemporary authors such as John Gerard and Nicholas Culpeper, their modes of reading could not be more different. Where Freke ruminated, digested and abstracted from Gerard's large tome, Boscawen made practical lists from Culpeper's *The English Physitian*. Secondly, the article shows that both supplemented their herbal reading with a range of other vernacular medical texts including printed medical recipe books, contemporary pharmacopoeia and surgical handbooks. Early modern English women's medical reading, I argue, was nuanced, sophisticated and diverse. Furthermore, I contend that well-informed readers like Boscawen and Freke made smart medical consumers and formidable negotiators in their medical encounters.

In 1615, Gervase Markham, having penned a number of successful advice manuals on husbandry and gentlemanly pursuits, turned his talents to instructing the women of England on how to go about their duties. *The English hus-wife* offers guidance on the ‘the inward and outward vertues which ought to be in a complete woman’. Significantly, in this manual for the ‘complete woman’, Markham begins not only with instructions for ‘inward vertues of the minde’, but also with ‘general Knowledges both in Physicke and Surgery, with plain approved medicines for health of the house-hold, also the extraction of excellent Oyles for these purposes’.^1^ Physic was considered by Markham to be ‘one of the most principal vertues which doth belong to our English Hous-wife’. Accordingly, it was necessary for her to have ‘a physicall kind of knowledge’, to know

… how to administer many wholsome receits or medicines for the good of their healths, as wel to prevent the first occasion of sicknesse, as to take away the effects and evill of the same when it hath made seasure on the body.^2^

To aid women on this quest, Markham offers a long section of medicinal recipes purportedly taken from a private manuscript compiled by a lady known for her skills in these areas.^3^

Markham's call for early modern English women to equip themselves with considerable skills in physic makes continual appearances in other works of this kind throughout the seventeenth century. His contemporary, Richard Brathwaite, also touches upon women's roles as healthcare providers in his tract *The English Gentlewoman*. Brathwaite writes:

Some Bookes shee reads, and those powerfull to stirre up devotion and fervour to prayer; others she reads, and those usefull for direction of her houshold affaires. Herbals she peruseth, which she seconds with conference: and by degrees so improves her knowledge, as her cautelous care perfits many a dangerous cure.^4^

Books and reading both play a crucial part in the life of Brathwaite's ideal gentlewoman. They not only guided her spiritual self-improvement but also enabled her to manage better her household duties. For Brathwaite, medical knowledge is, partly at least, to be gained from books. Books which our ideal woman would not only read but ‘peruse’ – that is to study, to examine, or to go through each entry one by one, be they entries on herbs or ailments or medicinal recipes. The ideal woman would then further confirm her knowledge by ‘conference’ – that is by seeking counsel and by participating in active discussions on her newly gained knowledge. Here Brathwaite is not only setting out advice for what to read but also *how* to read.

Alas, while Brathwaite may be prescriptive on reading practices, he falls short of providing his female audience with a definitive reading list. This omission is remedied by a late seventeenth-century advice book *The Gentlewomans Companion; or, A Guide to the Female Sex.*^5^ Here medical skills are considered essential as ‘it is a very commendable quality in Gentlewomen, whether young or old, to visit the sick; so it is impossible to do it with that charity some stand in need of, without some knowledge in Physick, and the several operations of Herbs and Spices.’^6^ Like Markham, the writers of this tract also provide readers with a short section on the use of medical herbs and spices and a number of ‘Choice and Experimental Observations in Physick and Chyrurgery, such which rarely fail'd any who made trial thereof’.^7^ Interestingly for the scope of this essay, the section on medical advices ends with a list of suggested readings. The writers advise:

… you would do well to acquaint yourself with the Composition of Mans Body and the Diseases incident to every part; which you may gather from several Books of Anatomy, either that of *Dr Read*, or *Dr. Riolanus* … If you would know the nature of Plants, *Gerhard* and *Parkinson* write incomparably on that Subject, but if they are too bulky, and so may seem tedious, you may make choice of lesser Herbals, as *Adam in Eden*; or a small Manuel written by *Mr. Lovel* … If you would have a Salve for every sore…and a receipt for every Distemper, consult the general practice of physic, *Riverius* his practice of Physick translated by *Mr Culpeper*.^8^

The reading list put forward in *The Gentlewomans Companion* aptly reflects the kinds of medical books available on the shelves of booksellers.^9^ By the second half of the seventeenth century, English printers and publishers offered readers a rich array of texts priced to suit almost all budgets. The market was flooded with folio-sized translations of Continental medical greats such as Daniel Sennert and Felix Platter, short pamphlets detailing know-how to treat particular illnesses, first aid manuals, pharmacopoeias and dispensatories, general medical guides, surgical handbooks and more.^10^ In this period, London book producers also offered a number of titles specifically marketed towards female readers. Many of these, such as *A Closet for Ladies and Gentlewomen* or *The Queens Closet Opened* presented readers with medical, culinary and household recipes. Significantly, the producers of *The Gentlewomans Companion* did not just recommend only texts intended for a female audience but rather hefty herbals and weighty translations of works by continental university physicians Jean Riolan and Lazare Rivière.^11^ The advice for medical reading seems to be to read widely and broadly.

This article explores how women read medicine in early modern English households.^12^ It builds on and extends a number of cross-discipline historiographical areas. Historians of early modern medicine have long recognized that vernacular printed medical books constituted one avenue through which householders obtained medical information. Paul Slack's and Mary Fissell's masterful surveys of medical print in sixteenth- and seventeenth-century England have provided scholars with a clear idea of the range of cheap (and not so cheap) medical books available to readers and householders.^13^ From the work of scholars such as Andrew Wear and Elizabeth Furdell, we have a good sense of the different kinds of information put forward by the myriad of popular medical handbooks in the period.^14^ While our picture of what book producers offered readers is relatively clear, we still know little about how these books were consumed.^15^ This article intends to address this gap in the historiography with a particular focus on how women, as medical caregivers in many early modern households, might have read vernacular medical books. What kinds of books did they purchase, read and consult? What sort of note-taking strategies did they employ in their engagement with these texts? And finally how do their reading practices fit into their medical activities and their own inquiries on nature?

In concentrating on women's medical reading practices within domestic spaces, this article also adds to existing narratives of household medical practices that highlight the importance of activities such as nursing and caring, the dressing of wounds, the administering of foods and medicaments and the making of drugs.^16^ At the heart of this article is the contention that reading about medicine, and about nature more generally, needs to be considered alongside these activities.^17^ Concurrently, this article responds to recent research in the history of science arguing for the importance of reading and writing practices in the transfer and codification of early modern natural knowledge.^18^ As it will become clear below, the ways in which women read medical books encompassed a wide range of actions. These varied from text selection and collection, information assessment, knowledge categorization and classification to the careful writing, recording and archiving of one's own treasury of knowledge. Reading about nature, thus, comes hand in hand with writing about nature. Finally, I participate in the rich conversation, amongst historians and literary scholars, on the reading practices of early modern English women.^19^ My study adds to our understanding of the sorts of reading materials that fuelled the minds of early female readers and the diverse ways in which they might have approached their reading matter.

## Women as Healers, Women as Readers

Within the historiography on the reading practices of early modern women, there are already a number of instances of women reading medical books. In fact, some of the most familiar and well-studied examples depicting early modern women reading actually concern medical books. Anne Clifford is one of these cases.^20^ Clifford, Countess of Pembroke, Dorset and Montgomery (1590–1676), has left historians with ample information about her reading practices in a number of sources including personal journals, a commissioned portrait and surviving copies of her annotated books. As such, Clifford's reading practices have been extensively reconstructed by Heidi Braymen Hackel and others.^21^ Often featured at the centre of these studies is, perhaps surprisingly, not notebooks of reading notes or annotated books but a rather large painting: the ‘Great Picture’ of the Clifford family. The triptych is often attributed to Jan van Belcamp and was commissioned and created under the close guidance of Clifford in 1646. It depicts Clifford's immediate family in the central panel flanked by a young and a matronly Anne in the side panels. In both side panels, Clifford is seen surrounded by books, of which the titles of many are clearly visible.^22^ In commissioning her own portrait, Clifford gave meticulous instructions not only to the representation of her own body and apparel but also the representation of her intellectual self. Both the young and the matronly Clifford are shown to possess an interest in a range of topics from history to moral philosophy to literature to architecture to religion. Of interest to the arguments presented here is the volume on the bottom of a horizontal pile of books stored on the lower shelf above the young Clifford's head. The volume is clearly labelled ‘The Epitome of Gerard's Herball’. Rebecca Laroche has argued that the use of the term ‘epitome’ to describe the book indicates that Clifford is not here citing a printed copy of Gerard's popular text but rather a handwritten manuscript notebook of reading notes taken from the tome.^23^ Clifford's decision to describe the volume as an epitome suggests that the work is a personalized summary or extract. The production of this work undoubtedly required both the perusal of the text and conference with others. Consequently, in Clifford's portrait, we see not only a visual record of an early modern gentlewoman reading a herbal but also reading it within the specific framework advocated by Brathwaite.

Anne Clifford was, of course, not alone in reading and extracting information from a herbal text. Not surprisingly, herbals were one of the main sources of information for home-based medical practices and it appears that herbals were fairly common reading matter for early modern women. Laroche provides us with twenty-four examples of early modern female readers' engagements with herbal texts both through inscribing and annotating in printed books and in extracting and recording their reading notes in manuscript notebooks.^24^ These include Grace Mildmay who read William Turner's herbal with her governess Mistress Hamblyn and Margaret Hoby who had a herbal read aloud to her by one of her servants.^25^ In particular, Clifford was in good company in her decision to extract from Gerard's herbal. In the archives, we often find selections of Gerard meticulously copied in contemporary manuscripts of medical reading notes.^26^ Tellingly, these are almost always extracts rather than mere copies and are thus records of readers' selection and, at times, reorganization of information. Gerard's tome, a richly illustrated folio-sized book of over a thousand pages, must have been a costly purchase. Perhaps due to the high cost of production, the book was also only issued in three editions and was probably hard to obtain by the later seventeenth century. These two factors undoubtedly encouraged large-scale summarizing, extracting and copying from the work. Of course, Gerard's book was not the only popular herbal text in the period. Nicholas Culpeper's *The English Physitian* also enjoyed a similar level of popularity. Issued in cheap octavo with a densely typeset text and no illustrations, Culpeper's work was also a favourite amongst medical readers and note-takers.^27^ In the remainder of this article, I explore the medical reading practices of two gentlewomen: Elizabeth Freke and Margaret Boscawen. Both of these women lived in large rural estates, were avid readers of books on health and medicine and left significant archives. I begin first by analysing how they read their herbals – one favoured Gerard and the other Culpeper – and end by marvelling at their wide-ranged medical reading practices.

## Elizabeth Freke and Her Abstract of Gerard

Elizabeth Freke (1642–1714) is, perhaps, now best known to historians as the author of two detailed sets of remembrances and as an enthusiastic producer and hoarder of medicines.^28^ However, her personal papers reveal that she was also an avid reader, note-taker and book collector. Freke's three surviving notebooks are filled with medical recipes, reading notes taken from medical, history and geography books and a detailed household inventory.^29^ The inventory, written into the notebook on 15 October 1711, includes a listing of her substantial library.^30^ Numbering over one hundred titles, the book collection was stored in two separate rooms in her house at West Bilney, Norfolk. In the downstairs closet, Freke kept books on religion, history, medicine and gardening in a ‘deep deale box by the fireside’.^31^ Above the hall in the upstairs closet, she kept in a ‘great chest’ what might be seen as the early modern equivalent to coffee-table books: Ogilby's lavish folio-sized tomes on China, Africa and America, his edition of Homer's Illiad and a myriad folio-sized history and law books.^32^ The pairing of reading notes and a book inventory makes Freke's archive an ideal case study for explorations into early modern reading practices.

Of Freke's three surviving notebooks, the volume most relevant to the present discussion is a thick vellum-bound folio-sized book containing a variety of information. Like many early modern miscellanies, the book was used from both ends.^33^ Culinary information was entered in the front of the book and, after the book was turned upside down, medical information was written in the back of the book. Sandwiched in between are medical reading notes, remembrances, copies of letters, the abovementioned inventory and other notes of rents and financial memoranda. Freke's reading notes are located in the ‘medical section’ of her notebook, immediately following her recipes for various ailments. These notes can be divided into five sections. The first is an extract out of her sister Austen's recipe book.^34^ The second, taken out of Nicholas Culpeper's translation of the *Pharmacopoeia Londinensis*, is titled ‘off seeds and grains’ and ‘some receits of short remembrance for my own use’.^35^ The third is her abstract of Gerard's herbal, followed by another selection of notes from Culpeper.^36^ The final section, titled ‘some short receits off remembrance for my use’, consists of notes taken from two contemporary pharmacopoeias intermingled with miscellaneous medical recipes from other sources.^37^

Within these copious reading notes, by far the most ink and paper are devoted to notes taken from John Gerard's *The Herball or Generall Historie of Plantes*. These, most likely taken from the enlarged second edition of the work, span over fifty-six folios and are accompanied with a detailed index for ease of information retrieval.^38^ With the first entry written on 5 February 1699/1700 and the last note not penned until 22 March of the same year, the project took Freke almost two months to complete.^39^ Freke referred to her notes from Gerard in two ways. In her inventory, she described it as an ‘abstractt of Gerralds herbal of my wrightine’. However, at the beginning of the actual notes, she titled it ‘A Collecttion of Receites taken out of Gerards Herball by me Eliz: Freke and for my owne use and Memory Abstracted 1699/1700’ suggesting that even though Gerard's work is not primarily a book of medical recipes, Freke regarded it as such.^40^ Freke's engagement with Gerard's text is driven by this view and as we will see, through her note-taking practices, Freke transforms Gerard's descriptive prose into recipes for action.

Just how did Freke read her herbal? Analysis of Freke's abstract, which maintains Gerard's organizational structure of three books and individual numbered entries, suggests that, despite the numerous indices in the printed work, she read through Gerard's work linearly and, impressively, pretty much from cover to cover. Her interaction with the work was, thus, less a hunt for specific information but rather more a steady browsing driven by her own personal interests. However, Freke's notes appear to be the result of not just one but repeated readings of the text. Within the listing of plant entries and her record of individual plant virtues, Freke's notes often differ from Gerard's text in the sequence used to list the information.

In these cases, she read ahead, skipping particular entries and then backtracked and took additional notes on the initially passed over information. If we further compare Freke's abstract to Gerard's text, it is clear that the notes rarely corresponded to the printed text word for word. Rather, Freke summarized in parts, rephrased in others and frequently combined Gerard's separate points into one entry. These notes took her the better part of a winter to complete because they involved multiple readings of the printed text, digestion of the information contained within and finally, selection and extraction of individual herbal entries.

After all her work, it is not surprising that Freke viewed her ‘abstract’ as a new, stand-alone text. It may have been copied in the same notebook as her remembrances and recipe collection but within her book inventory, it is listed as a distinct entry alongside other medical and religious tracts. As an independent text, Freke's abstract also came with its own new paratextual apparatus: an index, entry numbering and pagination. Eschewing Gerard's chapter and page numbering, Freke used her own system of entry numbers in both the main text and the index. Her decision to omit references to Gerard's work suggests that she did not plan on rereading or revisiting the printed book. For Freke, her own abstract contained all the information she needed and in her mind these reading notes have the same standing as other printed vernacular medical books. If we return to Anne Clifford and the display of her epitome of Gerard in the family triptych, it may be that Clifford too viewed her copy of Gerard along the same lines as Elizabeth Freke.

Freke's highly selective abstract reflects both her practical needs and her personal curiosities. For example, in the first section of Gerard's work, she only took notes on forty-seven out of a hundred and eighteen herb entries. Freke tended to select and note down herbs that had clear medicinal functions and omitted entries on ornamental plants.^41^ This drive for practical medicinal information can be further detected within the individual entries. As common in herbals, each entry in Gerard's work consisted of several sections: ‘the description’, ‘the place’, ‘the time’, ‘the name’, ‘the temperature’ and ‘the virtues’. The first three sections contain information that aids the reader to identify the plant in question. That is, a description of the plant's physical form and information on the general location and time of year during which gatherers can harvest the herb. Interestingly, and tellingly of knowledge and *materia medica* transmission across Europe, Gerard provides the reader with the name of the herb in a number of languages including Greek, Latin, Italian, Spanish, French, Dutch and English. Freke, for the most part, did not choose to record any of this information in her ‘abstract’. Rather, she concentrated on sections that describe the temperature or the complexion of the herb and its useful virtues suggesting that she already possessed the knowledge to identify the various medicinal plants.

A comparison of the first entry ‘meadow grass’ serves as a useful illustration of Freke's selective note taking. Gerard's entry on meadow grass is around one and half folio-sized pages long and comprises a woodcut image of the plant and the various sections described above.^42^ In contrast, Freke's entry is only four lines long and abstracts solely from the ‘virtues’ section.^43^ Within the virtues section itself, Freke chose to record four out of the five entries, omitting the cure for worms in children. It may be that as an elderly widow with a grown-up son and no grandchildren, Freke did not feel the need to scribble down cures for childhood ailments.^44^ Significantly, Freke also failed to note down the authorities ascribed to the different medicinal virtues. Gerard carefully informed his readers that Galen was the author who recommended the use of grass roots to ‘glue’ together wounds. Likewise, he notes that it was Jean Fernel who claimed that grass helped ‘obstructions of the liver, reines and kidnies and the inflammation of the raines called *Nephritis*’.^45^ Freke did not consider either of these two attributions worthy of copying. Aside from a handful of isolated instances, this kind of omission continued throughout her notes suggesting that for Freke, Gerard, as the compiler of this information, remained the central authority.^46^

Aside from impressing her interests on Gerard's text via a process of information selection, in a small number of entries, Freke also interpolated additional virtues and uses for the herbs and further clarified production methods with knowledge gained through her own medical practice. Her entry on couch grass is a good illustration of this point.^47^ Firstly, Gerard titled his entry ‘Of couch-grasse, or dogs-grasse’ and provided a third name, quitch-grass, for the plant under the section on naming. Freke titled her entry ‘Of Squitch grasse or couch grass’ suggesting that while Gerard considered ‘quitch-grass’ as merely an alternative name, for her this was the main name associated with the plant. Freke's entry summarized three out of four points in the printed work; though with subtle changes to the text. Where Gerard recommended the herb as one useful to provoke urine ‘gently’, Freke changed this to ‘gallently’ and where Gerard suggested that the herb ‘driveth forth gravell’, Freke chose instead to write that it ‘easeth the kidneys opressed with Gravell’. Additionally, Freke added ‘lett sick as are opressed with these diseases drink a draught of white wine boyled with these roots being first bruised for their Mornings Draught & Iff they find ease Lett them thank God, iff nott Blame mee’. Freke's willingness to take full responsibility for this recipe suggests that, even if the know-how was not of her own devise, she was confident of its efficacy. It may be that she has seen this medicine work first hand or has personally experienced the effects. The notes presented here thus reflect practices of both reading and observation.

The couch grass entry is merely one of many modified by Freke. In the entry for the ‘Golden Lungwort’, a herb recommended by Gerard as a medicine against whitlows and diseases of the lungs, Freke expanded this to include ‘hoarseness, coughs, wheezings, and shortness of Breath, Pthisicks, or Ulcerations of the lungs’. She also gave additional instructions that one ‘may boyle itt in hysop watter or coltfoot watter’.^48^ A final example is the entry on ‘Dane Wort or Wall-wort or Dwarf Elder’.^49^ In point ‘A’ of the ‘Vertues’ sections, Gerard wrote: ‘The roots of Wall-wort boiled in wine and drunken are good against the dropsie, for they purge downwards watery humours’. Freke added to this by writing:

… the roots of Dwarfe Elder are as Gallant a purge for the Dropsey as any under the sun, as [h]as bin offten proved by the never dying D^r^octter Buttler of Cambridg) you may take a dram or two drams att the Time (Iff the patientt be stonge) In white wyne or you may boyle the Roots in white wyne and drink itt against the Dropsey wch purges downwards watery humors.^50^

Here Freke not only provided detailed information on dosage but also linked the recipe to a well-known popular medical figure.^51^ In all these cases, Freke's own information on these herbs were interspersed with text copied from Gerard's book suggesting that, while reading and copying from Gerard's texts, Freke consulted other sources, be they other books or other experts or her own experience with the herb, to create a customized abstract. Elsewhere in her reading notes we see Freke intermingle information taken from printed pharmacopoeias with instructions provided by her sister Lady Norton and her niece Lady Getting.^52^ Norton's and Getting's offerings usually addressed the same ailment and subject matter as the notes taken from the printed text suggesting that they were results of conversations about a particular ailment initiated by Freke's reading. Here, Freke and her sisters are following Brathwaite's advice and seconding their reading with conference.

Elizabeth Freke's abstract of Gerard provides us with several lessons on medical reading. Firstly, it serves as a gentle reminder that modes of reading, even when it involves only one reader, can be nuanced and varied. Gerard's large tome was conceived as a reference guide. The inclusion of multiple indices sorting the book contents by Latin and English plant names and by ailment or disease encouraged readers to dip in and out of the book. Like the concordances described by Peter Stallybrass, this book was designed to be read discontinuously allowing readers like Freke to gear their reading towards their medical practices.^53^ Yet, Freke's reading for practice was not a hurried consultation of indices or a hunt for particular rare cures, rather it was a slow process of repeated readings, conversations and digestion. That is, it encompassed many of the characteristics of a mode of reading focused on rumination and reflection recently described by Jennifer Richards.^54^ At the same time, Freke's description of Gerard's herbal information as ‘receipts’ emphasizes the practical nature of her reading. The reading practices here are, thus, multifaceted and mixed. Secondly, Freke's reading process reminds us that reading was not a passive process or a simple, single linear knowledge transfer. Freke's abstract is not only based upon Gerard's text but rather also draws upon her trusted community of knowers, her extensive library and her own experiences and observations. While it might resemble a set of readings notes and so tempt historians to interpret it as a merely record of her engagement with one text, in reality, the notes present a new set of information constructed out of an interwoven process of reading, writing and face-to-face conversations and hands-on experiences. They are a record not only of knowledge consumption but also of knowledge production.

## Margaret Boscaswen's Useful Lists

A few decades before Freke began her abstract of Gerard, another gentlewoman, Margaret Boscawen (d. 1688) also studied and took notes from an herbal. Margaret was married to Hugh Boscawen (1625–1708) who, on and off, served as Member of Parliament for Cornwall, Tregothnan and Grampound between the late 1640s and 1701.^55^ The Boscawens lived in Tregothnan, Cornwall and were a well-to-do Cornish family who had considerable power within the county.^56^ Together Margaret and her daughter Bridget Fortescue, who inherited Margaret's papers, left a considerable archive of medical and natural knowledge with a number of different bound notebooks and a flurry of loose leaves.^57^ Within this complex archive, two slim volumes, mostly in Margaret Boscawen's hand, shows us how she read. One, an almost folio-sized notebook, was dedicated to medical information and medical recipes and another tall, slim notebook of only twelve folios was used to record information on herbs. Both volumes are revealing of Boscawen's reading practices and engagements with contemporary printed books.

In this section, I will focus on analysing the various ways Boscawen read her herbal. The herbal in this case was not John Gerard's luxurious tome but rather Nicholas Culpeper's *The English Physitian Enlarged. Or An Astrologo-Physical Discourse of the Vulgar Herbs of this Nation*.^58^ Seventeenth-century English readers had a wide choice when it came to herbals. Earlier works by William Turner and John Gerard continued to circulate and added to these were new titles by authors and compilers such as John Parkinson, William Cole and Robert Lovell and others.^59^ Within this book market, Culpeper's herbal was a huge publication success. By N. F. L. Poynter's count, more than a hundred editions had been printed by the 1960s and new editions continue to be issued.^60^ Culpeper himself was well aware of the competition in herbal titles and, in his letter to the reader, carefully outlined the various distinctive characteristics of his own offering. According to Culpeper, his work was novel in three aspects. Firstly, he focused on local English plants. Secondly, he provided ‘a Reason for every thing that is written, whereby you may find the very Ground and Foundation of physic, what to do and wherefore you do it’.^61^ Finally, his work provided explanations of disease causation and therapies within an astrological medical framework.^62^ With the surviving evidence, it is difficult to ascertain exactly why Boscawen chose Culpeper's work over others. Her remote Cornish location might have meant that local English plants were more readily available. It is likely that Boscawen was receptive to astrological medicine. That said, from the 1653 edition onwards, Culpeper's work was issued in octavo with no illustrations, and so it might also have represented the more affordable option.

Earlier in this article, we were introduced to Elizabeth Freke who took pains to create detailed, meticulous reading notes from John Gerard's enormous herbal. Margaret Boscawen's interaction with Nicholas Culpeper's equally rich book takes a somewhat different turn. Within her larger medical notebook, information taken from Culpeper's text appears on two separate sections. First, the references appear on a page with four columns showing medicines and ‘things’ good for the head, the heart and the spleen, the stomach and the liver. Under the column headed ‘Madisons Good for ye head’, Boscawen copied relevant information from a number of entries in *The English Physitian Enlarged* including information on rosemary, beet, ducks meat, costmary, dwarf elder, fetherfew.^63^ These are interspersed with remedies for ailments of the head gleaned from another title by Culpeper, *Culpeper's Last Legacy*.^64^ The herbs, with the exception of rosemary, are listed in the order they appear in Culpeper's index (under the entry on ‘headaches’), suggesting that Boscawen used this information retrieval aid to identify and make her selection. The other columns on the page were filled with medical information gained by contemporary recipe donors. One of the recipes, ‘For a Rhume in the Stomack,’ seems to have been collected in September 1673 from a yet-to-be identified ‘B. T.’. Other recipes are attributed to a Miss Plant and a Miss Jean Finnes who both contributed a number of recipes to the collection. Boscawen's tabular arrangement of medical information and recipes is unusual as are her choice of headings. If one assumes that by ‘head’ Boscawen is referring to the brain, it is likely that she is using the Galenic principle members – heart, brain and liver – as her organizational categories.^65^ Within the schema, the inclusion of the stomach and spleen is somewhat unusual. One might infer that Boscawen is here imposing her own ideas of the body upon the traditional Galenic framework. By juxtaposing medicines and ‘things’ for the three principal members (or perhaps five members in her own schema) which govern the organs and functions of the whole body, Boscawen encapsulated and visually represented health information for the human body on a single page.

Culpeper's *English Physitian Enlarged* appears again a few pages further on in her notebook. In this instance, she transformed Culpeper's alphabetically organized plant information into a collated list of distilled waters gathered from a number of different printed medical sources.^66^ The highly abstracted notes, often consisting of only a herb name or recipe title, paired with page numbers suggest that Boscawen saw her list as an external, personalized, customized index to the texts. The list referred to instructions to make remedies for a range of different ailments from well-known panaceas such as *aqua vita* to medicines for sore eyes. Here, the seemingly disparate information is united by their use of a similar methodology of production: distillation. Tellingly of Boscawen's own preoccupations, she noted alongside some of the titles, the specific type of still needed and the time of year recommended for the production of each medicine. Further emphasizing the list's function as a memory aid, in one entry Boscawen wrote: ‘How to make aqua composite: for the collik and stone for which I must remember to have some strong ale one month old: 137.’^67^

References to Culpeper's herbal can also be seen in Boscawen's ‘plant book’. The notebook is filled with headings such as ‘Roots to be saved’, ‘what Flowers should be saved and when yt time to gether’, ‘Flowers that must be gathered in May In Aprill’, ‘what seeds are to be saved’, ‘what syrups to make’, ‘what waters should be stilled’, ‘what water must be stilled in May and Jun’, ‘what Roots I must send for to London’ and ‘what Hearbs should be dryed’.^68^ Under each of these headings is a list of plant names. For some of these entries, Boscawen provided a book title written in shorthand and a page number.^69^ Most of these citations refer to Culpeper's *The English Physitian Enlarged.*^70^ At times, Boscawen added a few lines of additional information. This ranged from wood betony described as ‘for great use’^71^ to a short recipe in the case of wormwood flowers^72^ to more precise indications on when a plant must be gathered.^73^ The majority of the entries are very short with the longest entry no more than ten lines long. Boscawen's plant book reads very much like a curious hybrid of a collection of ‘to-do lists’ and an external personalized index to Culpeper's work. Her lists reminded her what needed to be accomplished at different times of the year and provided her with an immediate reference to more detailed information on particular *materia medica*. One wonders whether these lists also served as a simple inventory of the different roots, flowers and dried herbs stored away by Boscawen each year. If so, these lists would have performed the dual function of tracking both information and objects.

To create her lists, Boscawen extracted information from Culpeper's alphabetically organized knowledge schema and imposed her own categorization system. If Culpeper organized his plant knowledge to ensure readers' ease of information retrieval, Boscawen's system reveals her need to ‘get on top of’ her housewifely tasks – gathering herbs and flowers, preserving roots and seeds, distilling cordial waters and making syrups. The myriad of headings in Boscawen's plant book not only reminds modern readers of the large number of complex and multi-faceted tasks seen within the purview of household medicine but also functioned as a record of those labours. Undoubtedly, in large affluent households such as the Boscawens, the housewife's tasks are more managerial than hands-on, and, thus, we might also choose to read these lists as information and task management tools shared between Boscawen and her helpers. They also highlight the importance of seasonality and how early modern home-based medical practices were, to a certain extent at least, tied to the land. The lists also remind us that early modern medicine producers needed to plan ahead to both ensure availability of seasonal plant materials and to account for the long time frame required for the production of particular medicines.

Consequently, Boscawen's reading of Culpeper and her note-taking practices were part and parcel of her duties as an early modern housewife and healer and need to be seen alongside other medical tasks. Yet, in another way, Boscawen's lists speak of more than just utility. Each of these lists is the result of Boscawen's application of a different search criterion on Culpeper's text and a record of the various ways Boscawen used plant knowledge. By re-organizing the knowledge by task or by body part or type of medicament, Boscawen imposed her own system of knowledge categorization upon Culpeper's text. Far from passively reading printed books, Boscawen was re-imagining the knowledge contained within.

Like Freke's abstract of Gerard's herbal, Boscawen's reading of Culpeper's herbal is not a straightforward summary or scribal copy of the printed text. Both women engaged critically and selectively with their texts. Their notes also show us that, despite reading the same textual genre, they approached and created their medical treasuries in a vastly different ways. Where Freke strove to create her personalized stand-alone abstract of Gerard's expensive tome, Boscawen intended her notes to be used in conjunction with her medical library. Issues of access may account for these differences. For Freke quite plainly did not have continuous access to Gerard's text, whilst Boscawen's notes were clearly part of a larger medical library or archive comprising of both manuscript and printed books. However, other circumstances relating to access might also come into play. While Freke's residence, West Bilney, located in rural Norfolk, was within easy travelling distance to King's Lynn, Norwich, Newmarket and Bury St Edmunds, Freke herself was a frequent traveller and spent significant amounts of time visiting spa towns such as Tunbridge Wells and going to London for business and personal reasons. While housewifely medical tasks were a central part of her life, she also had continuous access to commercial medical services.^74^ Tregothnan, the Boscawen family seat, is located on the remote east Cornish coast near Truro and Falmouth. The Bocaswens were involved in the Cornish tin mines and, indeed, lived in mining country. When their kinswoman Celia Fiennes visited in 1698, she described how on her journey south, she did not travel on a coach road from the time when she left Exeter until she was around three miles out from Tregothnan.^75^ Tregothnan's relatively remote location must have encouraged Margaret Boscawen to cultivate her kitchen garden and to grow many of the plants and herbs required by the household for both food and medicinal purposes. Indeed, Fiennes's account of her visit to Tregothnan provides a brief sketch of the extensive gardens in the property which included a kitchen garden with paved gravel walks and boxed squares full of shrub-trees and an orchard filled with different fruit trees.^76^ Seen within this context, it is not surprising that, for Boscawen, those concise, task-orientated lists were a necessary part of estate management.

## Beyond Herbals: Women Reading Medicine

Thus far, my discussion has centred on our readers' engagement of herbals and plant information. However, both Freke and Boscawen were avid readers and their medical notebooks reveal that they consulted concurrently a wide range of vernacular medical texts. As financially comfortable gentlewomen, Boscawen and Freke were able to access the blossoming English medical book market and they owned six and eight printed medical books respectively. We have little additional information on the Boscawen family library but we know that in Elizabeth Freke's case, her medical books constituted just a small portion (less than a tenth) of her library, which consisted largely of religious, legal and history books. Interestingly, while on a number of instances, Boscawen records medical information gathered in the 1670s and 80s; her medical book collection appears to have been amassed in the mid 1650s. Freke, on the other hand, bought her medical books over a twenty-year period from the late 1670s to the late 1690s. The collections of both women covered four main areas: pharmacopoeias, contemporary medical recipe collections, general medical guides and herbals. In addition, both referred to and took notes from at least one manuscript book. Boscawen refers to ‘my brother Charles['s] book’ and Elizabeth Freke copied a large section out of her sister Austen's book.^77^ In both cases, manuscript and printed works were cited in similar ways, reminding us that, for early modern readers, the boundaries between the two media were fluid or in some cases non-existent.

Of our two readers, it is perhaps fair to say that Boscawen's tastes were a little more conservative than Freke's.^78^ Her choice of medical reading material includes many titles on the 1650s medical ‘bestseller lists’. These included three well-received works by Nicholas Culpeper: *The English Physitian Enlarged*, *Culpeper's Last Legacy* and his translation and edition of the *Pharmacopoeia Londinensis or The London Dispensatory.*^79^ Boscawen also consulted three printed recipe collections: Elizabeth Grey's *A Choice Manual*; *The Queen's Closet Opened* and Alexander Read's *Most Excellent and Approved Medicines* (London 1651).^80^ Printed recipe collections flourished in the 1650s.^81^ Many of these titles, like *A Choice Manual* and *The Queens Closet Opened*, were purposely targeted towards female readers. Whilst a number of titles, like these two, were supposedly penned by well-known aristocratic women, many others contained prefaces addressed to prominent women.^82^ These collections were widely used and it was not unusual for compilers, whether male or female, to copy recipes from printed remedy collections into their own. Aside from Boscawen, Anne Glyd and Anne Brumwich who both consulted and copied from Elizabeth Grey's *A Choice Manual* and the compiler of Wellcome Western MS 768 made a lengthy transcription from *A Rich Storehouse*.^83^

Elizabeth Freke also consulted a similar range of medical genres, however her choices are arguably more ‘edgy’. Like Boscawen, she consulted a number of herbals. In her case, these were John Pechey's *The Compleat Herbal of Physical Plants* (London, 1694) and John Gerard's *The Herball or Generall Historie of Plantes* (London, 1633). Freke also consulted a number of printed recipe collections, including the ambiguously titled ‘book of the family physition’^84^ and Nicholas Culpeper's *Culpeper's School of Physick* (London, 1659), a volume of miscellaneous writings collected together and published posthumously by his wife, Alice.^85^ Turning to pharmacopoeias, while Boscawen stuck with Culpeper's translation of the *Pharmacopoeia Londinensis*, Freke consulted Culpeper's translation plus the pharmacopoeias offered by the physician George Bates^86^ (augmented and translated by William Salmon^87^ ) and the Parisian apothecary Moyse Charas.^88^ Both of these works contain lengthy sections on chemical remedies and, in particular, Salmon's work, with his detailed commentary on chemical processes and terms, can been seen as an attempt to popularize iatrochemical ideas.

Freke's final reading choice undoubtedly also stemmed from her interests in iatrochemistry. Within her inventory of books is listed a ‘Book of Cirgrary of Colebatch’.^89^ John Colbatch was a controversial figure who repeatedly bumped heads with the Royal College of physicians.^90^ He first became known for his proprietary Vulnerary Powder and his Tincture of the Sulphur of Venus. In the 1690s he performed a series of public experiments using these medicines and published several treatises delineating his trials of the drugs and praising their merits. During the same period, he also published several tracts advocating his medical theory centred upon acids and alkalis. In her notebook, Freke referred to and copied information on two *materia medica* ‘asarabacca’ and ‘crocus metallorum’ from Colbatch's tract *A Physio Medical Essay Concerning Alkaly and Acid*.^91^ While Freke may have honed in on information concerning *materia medica*, her very purchase of Colbatch's text suggests that she possessed some interest in new medical ideas.

A theme running through both Boscawen and Freke's reading lists is the focus on practical medical guides. With the exception of Colbatch's work, both Boscawen and Freke's books are essentially guides to medicine production. Certainly, this must be in part due to supply, as the majority of the vernacular medical books coming off English presses in the period are practically orientated but I think that these book choices also speak to the aim of their reading. Both might have read for leisure elsewhere in library but here, they were reading for practice.^92^

Nor were they exceptional in doing so, for even though traces of early modern women's medical reading practices are hard to find, there are many other instances in the archives. In addition to the various examples provided about concerning women reading herbals, there is also documentation showing women readers exploring a variety of medical genres. For example, another mid-seventeenth-century gentlewoman, Elizabeth Sleigh, owned both Jacob Mosan's translation of Christopher Wirzung's *Praxis Medicinæ Uniuersalis; or A Generall Practise of Physicke* (London, 1598 and five subsequent editions) and the English translation of Jacques Guillemeau's midwifery text titled *Child-birth or, The Happy Deliverie of Women* (London, 1612 and 1635). 93 Other women reveal their book purchase choices by asserting their ownership within the pages of a book. For example, Frances Wolfreston signed her name in a copy of *The Books of Secrets of Albertus Magnus*, Mary Man wrote ‘Mary Man her book ano domany 1671’ on the title page of Girolamo Ruscelli's *The Secrets of Maister Alexis of Piemont* and Anne Coates wrote her name in a thirty-year-old copy of *The Queens Closet Opened*.^94^ Coates and Boscawen were not alone in consulting decades-old books. The Wellcome Library's copy of the 1651 edition of John French's *The Art of Distillation* contains a cornucopia of annotations and notes written in by Rebecca Tallamy and other members of the Tallamy family in the 1730s. In fact, running out of space in the margins and blanks of French's work, the Tallamy family decided to bind the printed book with an additional 140 blank leaves to allow them to further expand their collection of medical and culinary know-how.^95^ Thus, the printed medical book, once a conduit for medical knowledge, now becomes the receptacle. Like Boscawen and Freke, Rebecca Tallamy also read several medical books at the same time. References linked to several recipes suggest that the Tallamys were, at the very least, reading Nicholas Culpeper and William Salmon alongside John French.^96^

## Conclusions

By the mid seventeenth century, female readers in early modern English households fully utilized the offerings coming off the printing presses to extend, confirm and challenge their own medical knowledge. Not only did gentlewomen like Elizabeth Freke and Margaret Boscawen read a wide range of texts but they also engaged with these texts actively. To return to Brathwaite's terms, they perused, questioned and conversed about the information proffered in printed texts and re-codified the know-how to suit their own needs. Women's medical reading practices were, thus, sophisticated syntheses of a range of medical and natural knowledge circulating in a variety of media. Here, acts of reading and writing themselves are also ways in which informal medical knowledge is transferred and created. Boscawen's and Freke's active engagement with vernacular medical texts, in both manuscript and print, places them in the role of knowledge producers. Just as early modern natural philosophers might have relied on both readings and observations to understand the natural world, Boscawen and Freke combined time spent poring over books and time spent trying cures, planting herbs and making medicines.

The notebooks of Boscawen and Freke also highlight the divergent ways that were employed by early modern readers to seek out and appropriate natural knowledge. While we can paint both readers as elderly gentlewomen reading and healing in remote estates, their book choices and the ways in which they utilized their libraries were vastly different. Nowhere is this more evident than in their notes on plant information. Freke's stand-alone abstract of Gerard's herbal and Boscawen's useful lists taken from Culpeper's *The English Physician Enlarged* emphasize how individual readers approached the same textual genre in a myriad of ways. In addition, Boscawen's two sets of differing notes from *The English Physician*, the to-do lists in her plant book and the topical lists in her miscellany, remind us that a reader can also approach a single title in varying ways. My study of Boscawen and Freke's medical reading confirms recent arguments put forward by historians of reading on the need to study different modes of reading: acknowledgement of individual readers and the importance of reconstructing social, economic and political contexts for different sets of readers.^97^ Freke and Boscawen's personal requirements (based on needs and interests) for health-related information tailored their medical reading practices. These same requirements most certainly also affected the other ways in which these two women participated in contemporary medical markets. While there is little evidence to suggest that either offered their medical services or sold their homemade remedies commercially, both likely interacted with contemporary medical practitioners and medicine producers as active patients and smart consumers.^98^ The kinds of health-related knowledge gained by Freke and Boscawen via their reading would have deeply influenced their medical decision-making. Consequently, in a way, the stress placed by historians of reading on creating individuated narratives can also be extended to our studies of medical knowledge transfer and medical consumption. In recent years, historians of early modern medicine have worked to construct long narratives of medical consumerism and of the gradual commercialization of medical care in Britain and beyond.^99^ My studies of the Boscawen and Freke archive suggest that in sketching these sweeping histories, it pays to remember the quirks of individual actors. For sophisticated knowledge makers such as Boscawen and Freke must have made formidable negotiators in their medical encounters.

## Footnotes

1 Chapter heading for Chap. 1, Gervase Markham, *The English Hus-wife* (London, 1631), 1.

2 Gervase Markham, *The English hus-wife* (London, 1615), 4.

3 Gervase Markham, *Countrey Contentments, in Two Bookes: the First, Containing the Whole Art of Riding Great Horses in Very Short Time … The Second Intituled, The English husvvife* (London, 1615), sig. Q1v.

4 Richard Brathwaite, *The English Gentlewoman* (London, 1631) STC 3565.5, sig. Gg 1r.

5 This work is usually attributed to Hannah Wolley; however, Elaine Hobby has put forward convincing arguments to put the authorship of the work into dispute: *idem*, ‘A Woman's Best Setting Out is Silence: The Writings of Hannah Wolley’, in G. MacLean (ed.), *Culture and Society in the Stuart Restoration* (Cambridge, New York and Melbourne: Cambridge University Press, 1995), 179–200.

6 *The Gentlewomans Companion; or, A Guide to the Female Sex* (London, 1675), 161.

7 *Ibid*.,161–165 and 168–84.

8 *The Gentlewomans Companion; or, A Guide to the Female Sex* (London, 1678), 183–184.

9 Alexander Read, *A Manuall of the Anatomy of the Body of Man* (London, 1634 and many other editions); Johannes Riolanus, *A Sure Guide; or, The Best and Nearest Way to Physick and Chyrurgery* (London, 1657); John Gerard, *The Herball or Generall Historie of Plantes* (London, 1597, 1633 and 1636); John Parkinson, *Paradisi in Sole Paradisus Terretris. Or a Garden of All Sorts of Pleasant Flowers *…** (London, 1629 and many other editions); William Coles, *Adam in Eden: or, Natures Paradise *(London, 1657); Robert Lovell,* Pambotanologia. Sive Enchiridion Botanicum. Or a Compleat Herball *…** (London, 1659 and 1665) and Lazare Rivière, *The Practice of Physick* (London, 1655).

10 Mary Fissell, ‘Popular Medical Writing’, in J. Raymond (eds.), *The Oxford History of Popular Print Culture. Cheap Print in Britain and Ireland to 1660* (Oxford: Oxford University Press, 2011), 417–430 and *idem*, ‘The Marketplace of Print’, in M. Jenner and P. Wallis (eds.), *Medicine and the Market in England and Its Colonies c. 1450– c. 1850* (Basingstoke and New York: Palgrave Publishing, 2007), 108–132; Paul Slack, ‘Mirrors of Health and Treasures of Poor Men: The Uses of the Vernacular Medical Literature of Tudor England’, in C. Webster (ed.), *Health, Medicine and Mortality in the Sixteenth Century* (Cambridge: Cambridge University Press, 1979), 237–273 and Elizabeth Lane Furdell, *Publishing and Medicine in Early Modern England* (Rochester, NY: University of Rochester Press, 2002).

11 Jean Riolan the younger (1580–1657) taught at the University of Paris and was known for his anatomical textbooks. Lazare Rivière (1589–1655) taught at Montpellier and published a number of medical textbooks: Laurence Brockliss and Colin Jones, *The Medical World of Early Modern France* (Oxford: Oxford University Press, 1997), 100 and 151–3.

12 It is not my intention to suggest that women were the sole purveyors of domestic medicine. Elsewhere I have argued that early modern fathers, husbands, uncles and sons were also interested and active participants of home-based medicine and, of course, they were readers too. Elaine Leong, ‘Collecting Knowledge for the Family: Recipes, Gender and Practical Knowledge in the Early Modern English Household’, *Centaurus* 55 (2013), 81–103.

13 Slack, ‘Treasuries of Health’, Fissell, ‘Popular Medical Writing’ and ‘Marketplace of Print’ and Furdell, *Publishing and Medicine*.

14 Andrew Wear, *Knowledge and Practice in English Medicine, 1550–1680* (Cambridge: Cambridge University Press, 2000) and Furdell, *Publishing Medicine.* Furdell, in particular, provides a good overview of female printers and the production of medical print, Chap. 5.

15 There are, of course, exceptions but broadly speaking, aside from an article by Mary Fissell, most of the work on medical reading has been carried out by medievalists. Mary Fissell, ‘Readers, Texts and Contexts. Vernacular Medical Works in Early Modern England’, in R. Porter (ed.), *The Popularization of Medicine, 1650–1850* (London and New York: Routledge, 1992), 72–96. Medical reading in the medieval period has received considerable attention, see, for example, Peter Murray Jones, ‘Reading Medicine in Tudor Cambridge’, *Clio Medica* (1995), 153–183; and ‘Book Ownership and the Lay Culture of Medicine in Tudor Cambridge’, in H. Marland and M. Pelling (eds.), *The Task of Healing: Medicine, Religion and Gender in Early Modern England and the Netherlands 1450–1800* (Rotterdam: Eramus, 1996), 49–68; Monica Green, *Making Women's Medicine Masculine. The Rise of Male Authority in Pre-Modern Gynaecology* (Oxford: Oxford University Press, 2008); *idem*, ‘Books as a Source of Medical Education for Women in the Middle Ages’, *Dynamis: Acta Hispanica ad Medicinae Scientiarumque Historiam Illustrandam* 20 (2000), 331–369 and *idem*, ‘The Possibilities of Literacy and the Limits of Reading: Women and the Gendering of Medical Literacy in *idem*, *Women's Healthcare in the Medieval West: Texts and Contexts* (Aldershot: Ashgate Press, 2000), essay VII.

16 For a broader context of women's medical work in early modern Europe, see other articles in this volume and in the *Bulletin of the History of Medicine* special issue on ‘Women, Health and Healing in Early Modern Europe’, *Bulletin of the History of Medicine* 82 (2008), particularly the essays by Mary Fissell, Montserrat Cabré, Alisha Rankin and Elaine Leong. Additionally, for a German perspective, see, for example, Alisha Rankin, *Panaceia's Daughters: Noblewomen as Healers in Early Modern Germany* (Chicago: University of Chicago Press, 2013). For an Italian perspective, see amongst others, Tessa Storey, ‘Face Waters, Oils, Love Magic and Poison: Making and Selling Secrets in Early Modern Rome’, in E. Leong and A. Rankin (eds.), *Secrets and Knowledge in Medicine and Science, 1500–1800* (Aldershot: Ashgate Press, 2011), 143–163. Recent works on early modern Britain include Hannah Newton, *The Sick Child in Early Modern England 1580–1730* (Oxford: Oxford University Press, 2012) and Alun Withey, *Physick and the Family. Health Medicine and Care in Wales, 1600–1750* (Manchester: Manchester University Press, 2012). Pioneering works on the topic include Doreen Nagy, *Popular Medicine in Seventeenth-Century England* (Bowling Green, OH: Bowling State University Popular Press, 1988), Chap. 5 and Lucinda Beier, *Sufferers and Healers: The Experience of Illness in Seventeenth-Century England* (London: Routledge and Kegan Paul Ltd., 1987), Chap. 8.

17 While it is not within the scope of my study, I think that it is also important to recognize that reading is also a medical activity in another sense. That is, as literary scholars such as Jennifer Richards, Michael Schoenfeldt and Helen Smith have argued, the act of reading itself is an embodied practice. See, for example, Adrian Johns, ‘The Physiology of Reading’, in Marina Frasca-Spada and Nicholas Jardine (eds.), *Books and the Sciences in History* (Cambridge: Cambridge University Press, 2000), 291–316; Michael Schonefeldt, ‘Reading Bodies’, in Steven Zwicker and Kevin Sharpe, *Reading, Society and Politics in Early Modern England* (Cambridge: Cambridge University Press, 2003), 215–243; Helen Smith, *‘Grossly Material Things’. Women and Book Production in Early Modern England* (Oxford: Oxford University Press, 2012), Chap. 5 and Jennifer Richards, ‘Useful Books: Reading Vernacular Regimens in Sixteenth-Century England’, *Journal of the History of Ideas* 73 (2012), 247–271.

18 There is now a vast body of literature on this topic. Recent works include: Volker Hess and Andrew Mendelsohn (eds.), ‘Paper Technology in der Frühen Neuzeit’ *NTM. Zeitschrift für Geschichte der Wissenschaften, Technik und Medizin* 21 (2013); Lorraine Daston, ‘The Sciences of the Archive’, *Osiris* 27 (2012): 156–187 and Ann Blair, *Too Much to Know: Managing Scholarly Information before the Modern Age* (New Haven, CT: Yale University Press, 2010).

19 This is now a rich and active field and relevant works are too numerous to name here. Recent works include Julie Crawford, ‘Reconsidering Early Modern Women's Reading, or, How Margaret Hoby Read her de Mornay’, *Huntington Library Quarterly* 73 (2010), 193–224; Heidi Brayman Hackel and Catherine E. Kelly (eds.), *Reading Women: Literacy, Authorship, and Culture in the Atlantic World, 1500–1800* (Philadelphia: University of Pennsylvania Press, 2009); William Sherman, *Used Books: Marking Readers in Renaissance England* (Philadelphia: University of Pennsylvania Press, 2008), Chap. 3; Heidi Brayman Hackel, *Reading Material in Early Modern England: Print, Gender and Literacy* (Cambridge: Cambridge University Press, 2005); Victoria E. Burke, ‘Ann Bowyer's Commonplace Book (Bodleian Library Ashmole MS 51): Reading and Writing Among the “Middling Sort”’, *Early Modern Literary Studies* 6 (2001), 1–28 and David McKitterick, ‘Women and their Books in Seventeenth-Century England: The Case of Elizabeth Puckering’, *The Library*, 7th ser. 1 (2000), 359–380.

20 Jan van Belcamp (attributed), *The Great Picture*, 1646. The portrait, a large painting of Lady Anne and her family, was commissioned by Lady Anne and is now in the Abbot Hall Art Gallery in Kendal. The left panel shows Lady Anne at age fifteen standing in front of some of her prized possessions, including a volume of Gerard's Herbal. For information on the portrait, see G. Parry, ‘The Great Picture of Lady Anne Clifford’ in D. Howarth (ed.), *Art and Patronage in the Caroline Courts* (Cambridge: Cambridge University Press, 1993), 202–219.

21 See, for example, Brayman Hackel, *Reading Material*, 221–240 and Mary Ellen Lamb, ‘The Agency of the Split Subject: Lady Anne Clifford and the Uses of Reading’, *English Literary Renaissance* 22 (1992), 347–368.

22 For a transcript, see, Richard T. Spence, *Lady Anne Clifford: Countess of Pembroke, Dorset and Montgomery 1590–1676* (Stroud, Gloucestershire: Sutton Publishing, 1997), 190–191.

23 Rebecca Laroche, *Medical Authority and Englishwomen's Herbal Texts 1550–1650* (Aldershot: Ashgate Press, 2009), 17–18.

24 *Ibid*., Chaps. 2 and 3. A full list of the 24 examples is provided in Appendix B (171–4).

25 Linda Pollock, *With Faith and Physick. The Life of a Tudor Gentlewoman Lady Grace Mildmay 1552–1620* (New York: St Martins Press, 1993), 97; and Margaret Hoby, *The Private Life of an Elizabethan Lady: The Diary of Lady Margaret Hoby 1599–1605*, ed. J. Moody (Stroud: Sutton Publishing, 2001), 29. See also Crawford, ‘Reconsidering Early Modern Women's Reading’.

26 A number of men and women turned to Gerard for information, including Elizabeth Digby, George Noble, Joseph Brooker and an anonymous compiler whose collections are now in the library of the New York Academy of Medicine (hereafter NYAM): British Library (hereafter BL), Egerton MS 2197, Wellcome Library (hereafter Wellcome), Western MS 579, MS 1364 and MS 8575 and NYAM, MS ‘A Collection of Medical Remedies for Colic and other’.

27 See, for example, Wellcome, Western MS 4053 or National Library of Medicine, MS b 261.

28 See, for example, Raymond A. Anselment, *The Remembrances of Elizabeth Freke 1671–1714* (Cambridge: Cambridge University Press, 2001) and Melosina Lenox-Conyngham, *Diaries of Ireland: An Anthology, 1590–1987* (Dublin: Lilliput Press, 1998). For an account of Freke's activities as a medicine producer and her various drug-filled cupboards, see Elaine Leong, ‘Making Medicines in the Early Modern Household’, *Bulletin of the History of Medicine* 82 (2008), 145–168.

29 The Freke papers are now in the British Library (Additional MSS 45718–21). Add MS 45718 is bound in white vellum and contains a version of Freke's remembrances and her medical and culinary notes. Add MS 45719 is bound in brown wallpaper and presents a second neater copy of the remembrances. Add 45720 contains geographical and historical notes collected by Freke.

30 British Library, Additional MS 45718 (hereafter 45718), fols. 90r–94v.

31 For a discussion on early modern closets and, particularly their role as a space for storing valuables, see Lena Cowen Orlin, *Locating Privacy in Tudor London* (Oxford: Oxford University Press, 2007), Chap. 8.

32 For a detailed title listing with full notes, see Anselment, *The Remembrances of Elizabeth Freke*, 173–177.

33 For further information on this practice see: Jonathan Gibson, ‘Casting off Blanks: Hidden Structures in Early Modern Paper Books’, in James Daybell and Peter Hinds (eds.), *Material Readings of Early Modern Culture. Texts and Social Practices 1580–1730* (Basingstoke: Palgrave Macmillan, 2010), 208–228.

34 45718, fols. 244r–22r.

35 Ibid., fols. 221v–217r and 216v–214v.

36 Ibid., fols. 214r–158r, 157v–150r and 149v–141r.

37 Ibid., fols. 140v–121v. The sections copied out of the pharmacopoeias of Moyse Charas and George Bate are at fols. 132r–128r and 128r–125v. See below for further information on the two printed publications and the entries.

38 Both these editions are Thomas Johnson's enlarged versions of Gerard's original text: John Gerard, *The Herball or Generall Historie of Plantes* (London, 1633 and 1636) (hereafter Gerard 1633).

39 45718, fols. 214r–158r.

40 Ibid., fols. 91v and 214r. Freke was not the only note-taker who referred to their notes as ‘receits’. The compilers of Wellcome MS 8575 who extracted a number of entries from Gerard labeled their section as ‘These Recit/receits I did take out of Mr Gerarde Herball’ (fol. 170r).

41 In a few instances, Freke makes a record of Gerard's inclusion of these plants. For example her entry on the ‘Cuttchonelle’ or ‘scarlett oke’ ends with ‘the Greatt Scarlett oke, And the great Holme Oke are more for Beauty than Vertues’, 45718, fol. 173r.

42 Gerard 1633, 1.

43 45718, fol. 214r.

44 Freke's first grandson was born later in 1700.

45 Gerard 1633, 1.

46 For example, in her entry on Broom, she records that certain virtues are from ‘Mesue’, or in her entry on Rest Harrow, she references ‘Matthiolus’ but in the same entry, she does not note that other information was attributed to Pliny. The instances where she notes attributes are relatively rare and there does not appear to be any consistency or patterns behind these citations. Some of the authors are ancient authorities, others modern. In some instances, as in the case of Mattioli and the Rest Harrow, the authority is associated with reporting an actual case but in other instances the authority is linked with general information: 45718, fols. 174v and 174r.

47 45718, fol. 210v and Gerard 1633, 23–4.

48 45718, fol. 209v.

49 45718, fol. 169v and Gerard 1633, 1425–6.

50 45718, fol. 169v.

51 Thompson Cooper, ‘Butler, William (1535–1618)’, rev. Sarah Bakewell, in *Oxford Dictionary of National Biography*, ed. H. C. G. Matthew and Brian Harrison (Oxford: Oxford University Press, 2004); online edn., ed. Lawrence Goldman, http://www.oxforddnb.com/view/article/4217 (accessed February 2014).

52 For example, on folio 131v, is written three recipes for the eye. Two of these were taken out of Moyse Charas, *The Royal Pharmacopoea, Galenical and Chymical* (London, 1678) and one recipe is attributed to Lady Getting: 45718, 131v and Charas, *The Royal Pharmacopoea*, 230 and 234.

53 Peter Stallybrass, ‘Books and Scrolls: Navigating the Bible’, in Jennifer Andersen and Elizabeth Sauer (eds.), *Books and Readers in Early Modern England. Material Studies* (Philadelphia: University of Pennsylvania Press, 2002), 42–79.

54 Richards, ‘Useful books’.

55 ‘Hugh Boscawen I (1625–1701), of Tregothnan, Cornwall and Greek Street, Westminster’ in *The History of Parliament: the House of Commons 1690–1715*, eds. D. W. Hayton, Eveline Cruickshanks, Stuart Handley (London: Boydell and Brewer, 2002); online edition: http://www.historyofparliamentonline.org/volume/1690-1715/member/boscawen-hugh-i-1625-1701 (accessed February 2014).

56 It seems that Hugh Boscawen was a resident or a lodger on Greek Street in the late 1690s. Boscawen's status as a resident or lodger suggests that, unlike some Members of Parliament, Hugh Boscawen did not keep a full household in both London and Cornwall: ‘Greek Street Area: Portland Estate: Greek Street’, Survey of London: Volumes 33 and 34: St Anne Soho (1966), 170–190. URL: http://www.british-history.ac.uk/report.aspx?compid=41077 (accessed February 2014). For more information on the Boscawen family, see Alan Bryson, ‘Boscawen, Hugh (bap. 1578, d. 1641)’, in *Oxford Dictionary of National Biography*, ed. H. C. G. Matthew and Brian Harrison (Oxford: Oxford University Press, 2004); online edition., edited by Lawrence Goldman, January 2008. http://www.oxforddnb.com/view/article/53019 (accessed February 2014).

57 These papers evidently were passed onto Margaret Boscawen's daughter Bridget Boscawen Fortescue and are now part of the Fortescue family papers at the Devon Record Office. The three folders are Devon Record Office 1262M/FC/6–8. I am grateful to Anne Stobart for introducing me to the Fortescue archive. For more information on the Boscawen/Fortescue archive see, Anne Stobart, ‘The Making of Domestic Medicine: Gender, Self-Help and Therapeutic Determination in Household Healthcare in South-West England in the Late Seventeenth Century (Middlesex University, unpublished PhD thesis, 2008).

58 London, 1653. This is the second edition of Culpeper's wildly popular *The English Physitian. Or An Astrologo-Physical Discourse of the Vulgar Herbs of this Nation* (London, 1652). The combination of the numerous editions and Boscawen's sparse notes makes it difficult to identify the exact edition used. However, it appears that many of the page numbers quoted by Boscawen correspond to the 1653 edition. See below for details.

59 Agnes Arber, *Herbals. Their Origin and Evolution. A Chapter in the History of Botany 1470–1670* (Cambridge: Cambridge University Press, 1986), 284–285.

60 ‘Nicholas Culpeper and His Books’, *Journal of the History of Medicine and Allied Sciences* 17 (1962), 152–167.

61 Nicholas Culpeper, *The English Physitian. Or An Astrologo-Physical Discourse of the Vulgar Herbs of this Nation* (London, 1652), sigs. A2v–A3r.

62 *Ibid*., sig. A3r.

63 Devon Record Office 1262M/FC/6. Large receipt book. The book is unfoliated. The verso side of the folio is filled with medicines for the head. The four columns continue on the recto side of the next folio but the page is largely empty.

64 London, 1655.

65 Nancy Siraisi, *Medieval and Early Renaissance Medicine. An Introduction to Knowledge and Practice* (Chicago: University of Chicago Press, 1990), 107–109.

66 See the later section ‘Beyond Herbals: Women Reading Medicine’ in this article for further details on the various medical books listed in Boscawen's manuscripts.

67 Devon Record Office 1262M/FC/6. Large receipt book. For the use of lists as memory aids, see Valentina Pugliano, ‘Specimen Lists. Artisanal Writing or Natural Historical Paperwork?’, *Isis*, 103.4 (2012), 716–726.

68 Devon Record Office 1262M/FC/7, plant notebook (hereafter plant notebook), fols. 1v, 2r, 34, 4v, 6r, 8r, 8v, 9v and 10r. For a partial transcription of this manuscript see, Stobart, ‘The Making of Domestic Medicine’, 308–10.

69 By the seventeenth century, a number of shorthand systems were in use. For a contemporary summary and comparison of these systems see: Elisha Coles, *The Newest, Plainest and the Shortest Short-hand* (London, 1674). I have been unable to identify the exact system used by Boscawen but her notes most closely match that of Edmond Willis as described in *An Abreuiation of Writing by Character* (London, 1618). For an introduction to shorthand systems in early modern England, see Frances Henderson ‘Reading, and Writing, the Text of the Putney Debates’, in M. Mendle (ed.), *The Putney Debates of 1647. The Army, The Levellers, and the English State* (Cambridge: Cambridge University Press, 2001); ‘A Note on William Clarke's Shorthand’, in *idem* (ed.), *The Clarke Papers. Further Selections from the Papers of William Clarke* (Cambridge: Cambridge University Press, 2005), 375–382; and Adele Davidson, *Shakespeare in Shorthand. The Textual Mystery of King Lear* (Newark: University of Delaware Press, 2009), Chaps. 1 and 2. For women's use of shorthand in the period, and in particular to conceal information, see Margaret Ezell, ‘Domestic Papers: Manuscript Culture and Early Modern Women's Life Writing’, in M. M. Dowd and J. A. Eckerle (eds.), *Genre and Women's Life Writing in Early Modern England* (Aldershot: Ashgate Press, 2007), 33–63 (at 40–41).

70 Culpeper's *The English Physitian* has a long publication history. Boscawen's notes refer to the second expanded edition of the work first published in 1653 (Wing C7502).

71 ‘wood Betony of great use: [shorthand characters] 31’, plant notebook, fol. 1r. This corresponds to Culpeper, *The English Physitian Enlarged*, 31. In the printed text, Culpeper writes: ‘It is found by dayly experience to be good for many diseases.’

72 ‘Flowres of wormwood which is in Agaust: It is good to make a conserve of with Roem Rose-mary bloums and black thorn: of each a like quantity: halfe that quantely of safforne, boyle this in Renish wine, but put not in the safforne till it be almost boyled, this is the way to keep a mans body in health, [shorthand characters]: 376’, plant notebook, fol. 2r. This recipe can be found in Culpeper, *The English Physitian Enlarged*, 376.

73 See, for example, the entry ‘Borrage and Buglos: In May: but especially in Jun and July’ under the heading ‘Flowers that must be gathered in May [shorthand characters] in Aprill’, plant notebook, fol. 3r.

74 Leong, ‘Making Medicines in the Early Modern Household’. For an account of the many different kinds of medical services and goods purchased by Freke during her husband's last illness, see BL, Additional MS 45719, fols. 37v–40v.

75 In her account of her Cornish travels, Fiennes also mentions the hazardous ‘Cribly ferry’ crossing and travels over step stony hills: Celia Fiennes, *Through England on a Side Saddle in the Time of William and Mary. Being the Diary of Celia Fiennes*, ed. Emily W. Griffiths (London and New York: Field and Tuer, The Leadenhall Press *et al*., 1888), 216–217.

76 Fiennes, *Through England on a Side Saddle*, 220.

77 Devon Record Office 1262M/FC/6. Large receipt book and 45718, fols. 244r–22r.

78 Our view of Boscawen's reading material is, of course, somewhat coloured by the fact that our main source lies in her medical notebooks whereas Freke's book inventory gives us a rare glimpse of an early modern woman's library. A household inventory of Tregothnan survives but it dates to the beginning of the eighteenth century (after Margaret's death) and does not give specific information about books: The National Archives: Public Record Office, Chancery Masters' Exhibits C108/67.

79 Boscawen's reading notes refer to the 1654 edition of Culpeper's *Pharmacopoeia Londinensis or The London* Dispensatory (Wing C 7526) and the first (1655) edition of *Culpeper's Last Legacy* (Wing C 7518). Both these works were issued in multiple editions in the seventeenth century.

80 *A Choice Manual* was first published in 1653 and was reprinted multiple times in the seventeenth century. Boscawen consulted the expanded 1654 edition (Wing K312). *The Queens Closet Opened* was likewise a popular publication and Boscawen's reading notes matches to the 1655 (first) edition.

81 Fissell, ‘The Marketplace of Print’. Fissell shows that recipe books made up 22 per cent of the popular medical books printed between 1640–1740 116--7 (esp. Figs. 6.2 and 6.3).

82 In his preface to *The Widdows Treasure* (London, 1595), John Partridge clearly states that the work is meant for the private use of gentlewomen. Gervase Markham's *The English Hus-wife* (London, 1615) and *The Ladies Cabinet Enlarged* (London, 1655) have prefaces addressed to women.

83 BL, Additional MS 45196 and Wellcome, Western MS 160.

84 45718, fol. 91v. This likely refers to either Gideon Harvey's *The Family Physician, and the House Apothecary* (London, 1676) or George Hartman's *The Family Physitian* (London, 1696).

85 Freke's reading notes are mostly taken from two sections of the work. These are *Fragmenta Aurea*, a three part series with ‘golden’ sayings from famous personages such as Peter of Spain and Galen and *The Treasury of Life* which is a remedy collection organized according to ailments. *Culpeper's School of Physick* was re-issued three times in 1678 and a final edition appeared in 1696.

86 George Bates (1608–1669) was physician to Charles I and II and Oliver Cromwell, as well as being an active member of the College of Physicians. His *Pharmacopoeia Bateana* was published posthumously in Latin in 1688 with numerous later editions. The Latin version of the text contains Bate's prescriptions as edited by the apothecary James Shipton with five hundred of the physician Jonathan Goddard's chemical processes and explications under the title *Aracana Goddardiana*.

87 *Pharmacopœia Bateana: or, Bate's Dispensatory* (London, 1694 and many later editions). This is a translation, by William Salmon of the second edition of Bates' Latin text. Salmon also enlarged the text with his own additional commentary and recipes. The authorship of the recipes is clearly noted in square brackets throughout the text. The second edition, *Pharmacopœia Bateana, in English: or, Dr. Bate's Dispensatory Enlarged* (London, 1694), contained even larger numbers of recipes by Salmon, to the point where the recipes from both authors were similar in number. Freke most likely consulted the second edition of the work.

88 This is the English translation of Moyse Charas's *Pharmacopée Royale Galénique et Chimique* (Paris, 1676) which was first printed for John Starkey and Moses Pitt in 1678 as *The Royal Pharmacopoea. Galenical and Chymical*. Moyse Charas (1619–1698) was an apothecary and chemist in Paris in the mid seventeenth century. He once gained the title of ‘l'artiste démonstrateur au Jardin au roi’ and was apothecary to the Duke of Orléans. In the 1670s, he entered into a dispute with Franceso Redi over viper bites: Patrizia Catellani and Renzo Console, ‘Moyse Charas, Francesco Redi, the Viper and the Royal Society of London’, *Pharmaceutical Historian: Newsletter of the British Society for the History of Pharmacy* 34 (2004), 2–10.

89 This book description likely refers to one issue of Colbatch's collected works: *Four Treatises of Physick and Chirurgery* (London, 1698), or his *A Collection of Tracts, Chirurgical and Medical* (London, 1699, 1700 and 1704). Both of these contain the tract *A Physio-Medical Essay Concerning Alkaly and Acid* (1696) from which Freke copied; see below. Intriguingly, Colbatch shares Charas's interest in viper bites and penned in 1698 a tract titled *A Relation of a very Sudden and Extraordinary Cure of a Person Bitten by a Viper*. This is included in *A Collection of Tracts Chirurgical and Medical*.

90 Harold Cook, ‘Sir John Colbatch and Augustan Medicine: Experimentalism, Character and Entrepreneurialism’, *Annals of Science* 47 (1990), 475–505.

91 Freke, fol. 133r. Asarabacca is ‘The plant *Asarum Europæum*, sometimes called Hazelwort, used formerly as a purgative and emetic, and still as an ingredient of cephalic snuff’: *OED*. ‘Crocus metallorum’ was a substance ‘made of Antimony, Salnitre, (and some times fine Tartar) put to a Fusion and Detonation, the Faeces being separated produceth a fine Liver-coloured Crocus’: Jacob Berlu, *The Treasury of Drugs Unlock'd* (London, 1690), 36.

92 For more on ‘reading for action’ see Anthony Grafton and Lisa Jardine, ‘“Studied for Action”: How Gabriel Harvey Read His Livy’, *Past and Present* (1990), 30–78 and Crawford, ‘Reconsidering Early Modern Women's Reading’.

93 Wellcome, Western MS 751. This is the recipe collection of Elizabeth Sleigh and Felicia Whitfield. At the end of the notebook is a list titled ‘An inventory of the Lady Sleighs bookes. May 12, 1647’. Some fifty-two items are listed, most of which, like on Freke's list, are religious works.

94 Sig. A5r, Huntington Library, HN 17106; title page v on Folger Shakespeare Library, STC 297 and front flyleaf r in Folger Shakespeare Library, M 98. For addition information on Frances Wolfreston's reading habits, see Paul Morgan, ‘Frances Wolfreston and “Hor Bouks”: A Seventeenth-Century Woman Book-Collector’, *The Library*, 6th ser. 11 (1989), 197–219.

95 Wellcome, Western MS 4759. Interestingly, an annotator changed the publication date on the title page from 1651 to 1691, suggesting that there was some awareness that French's book required some updating (if only cosmetic) by the eighteenth century. Perhaps contrary to this, a number of the recipes written on the additional leaves are attributed to past well-known medical or political personages from the sixteenth and seventeenth centuries including Princess Elizabeth (fol. 129r), Queen Elizabeth (fol. 201v) and Dr Richard Lower (fol. 203r).

96 Wellcome, Western MS 4759, fols. 232v, 240r–241v and 243v. The recipe from Culpeper is titled: ‘A Diet Drink out of Culpeper’. The recipe could be copied from a number of works penned by Culpeper and, at present, I have not been able to directly link the manuscript reference to the printed work. The recipes labeled as ‘Salmon’ are taken from William Salmon's *Pharmacopoeia Londinensis or The New London Dispensatory* (London, 1678). They include ‘Quercetans Eye Water’, A Watter Against darkness of ye sight’, ‘Minsichts Blessed Laver’, ‘Syrup of Annis Seed’, Quiddony of Elder Berries and Spirit of Black Cherries'; Wellcome, MS 4579, fols. 240r–v and *The New London Dispensatory*, 720, 724, 610, 614 and 461.

97 Jennifer Richards and Fred Schurink, ‘Introduction: The Textuality and Materiality of Reading in Early Modern England’ and Fred Schurink, ‘Manuscript Commonplace Books, Literature, and Reading in Early Modern England’, *Huntington Library Quarterly*, 73 (2010), 345–361 and 453–69.

98 Margaret Pelling, *Medical Conflicts in Early Modern London. Patronage, Physicians and Irregular Practitioners 1550–1640* (Oxford and New York: Oxford Univeresity Press, 2003) and Sara Pennell and Elaine Leong, ‘Recipe Collections and the Currency of Medical Knowledge in the Early Modern “Medical Marketplace” ’ in Jenner and Wallis (eds.), *Medicine and the Market*, 133–152.

99 For example, Ian Mortimer, *The Dying and the Doctors. The Medical Revolution in Seventeenth-century England* (Woodbridge, and Rochester, NY: The Boydell Press, 2009) and Patrick Wallis, ‘Exotic Drugs and English Medicine: England's Drug Trade, c. 1550–1800’, *Social History of Medicine* 25.1 (2012), 20–46.

